# Audiological profile of patients with type 2 diabetes mellitus

**DOI:** 10.4102/sajcd.v71i1.1035

**Published:** 2024-07-31

**Authors:** Sakhile T. Nkosi, Vuyelwa Z. Peter, Jessica Paken

**Affiliations:** 1 Discipline of Audiology, College of Health Sciences, University of KwaZulu-Natal, Durban, South Africa

**Keywords:** audiological profile, type 2 diabetes mellitus, hypertension, hearing loss, adults

## Abstract

**Background:**

South Africa shows a high prevalence of type 2 diabetes with reported association with auditory dysfunction.

**Objectives:**

To describe the audiological profile of adults with this metabolic condition.

**Method:**

Employing a descriptive research design, 35 individuals with type 2 diabetes, selected through purposive sampling, underwent a basic audiological assessment in addition to extended high-frequency (EHF) audiometry, distortion product otoacoustic emissions (DPOAE) testing and neurological auditory brainstem response (ABR) test.

**Results:**

This study revealed a 31.4% prevalence of hearing loss with 81.8% being sensorineural in nature. Poor hearing thresholds were observed at 16 kHz (*n* = 19; 54.3%), 18 kHz (*n* = 24; 68.6%) and 20 kHz (*n* = 30; 85.7%) in the right ear and at 16 kHz (*n* = 20; 57.1%), 18 kHz (*n* = 24; 68.6%) and 20 kHz (*n* = 30; 85.7%) in the left ear. Absent DPOAEs were observed at 6 kHz (*n* = 20; 51.7%) and 8 kHz (*n* = 24; 68.6%) in the right ear and at 6 kHz (*n* = 17; 48.6%) and 8 kHz (*n* = 29; 82.9%) in the left ear, possibly indicating that type 2 diabetes specifically targets higher frequency hearing. The ABR results revealed a delayed absolute latency of wave III bilaterally (right ear –69%; left ear – 51%), suggesting an impact of this metabolic disease on retro-cochlear pathways.

**Conclusion:**

Hearing loss should be recognised as a comorbidity accompanying type 2 diabetes, which indicates the need for routine comprehensive audiological assessments to facilitate early detection and intervention.

**Contribution:**

The present findings have implications for audiology clinical protocols; diabetes related health policies and patient education.

## Introduction

Diabetes is a significant public health concern (International Diabetes Federation [IDF], 2021). In South Africa, an estimate of about 4.2 million adults (10.8%) between the ages of 20 and 79 years live with diabetes, with a projected proportion of undiagnosed cases of approximately 1922.2 (45.4%) (IDF, 2021). Without proactive intervention to prevent this disease, this trend is predicted to double by 2045 (IDF, 2021). With the increase in new diabetes cases, it is critical to explore the link between diabetes and hearing impairment.

Hearing impairment in diabetes may occur because of multisystem complications caused by the disease (Kibirige et al., 2019). Despite extensive literature confirming the association between diabetes and hearing loss, there is still some controversy surrounding this relationship (Akinpelu et al., 2017; Hlayisi et al., 2018; Sameli et al., 2017). This controversy is seen in the reported variation in diabetes-related hearing impairment prevalence worldwide. A study conducted in the United States (US) reported a prevalence rate of 13.1% (Kakarlapudi et al., 2003), while in Iran, a rate of 45% was reported (Mozaffari et al., 2010). The highest prevalence rates were observed in India and South Africa at 78.2% and 55.0%, respectively (Bhaskar et al., 2014; Hlayisi et al., 2018). There are various factors that could contribute to the aforementioned variation in hearing loss prevalence. Such factors may include diverse study designs, sample size composition, audiologic assessment methods used, hearing loss classification methods and presence of comorbidities (Hlayisi et al., 2018; Pillay et al., 2021; Sameli et al., 2017). It was noted that the majority of the studies used different threshold cutoff to determine the presence or absence of hearing loss. The use of pure-tone hearing threshold average (PTA) with different normative values of either 15 dB HL or 25 dB HL was evident in studies conducted by Thimmasettaiah and Shankar (2012) and Agarwal et al. (2013), whereas Hlayisi et al. (2018) used conventional pure-tone average (CPTA) and high-frequency pure-tone average (HPTA), thus contributing to the variation in hearing loss prevalence. While this difference in the hearing loss classification may result in variation in prevalence, co-morbidities such as hypertension may also be a contributing factor. It is important to note that diabetes and hypertension are inextricably linked diseases, and the prevalence of hypertension is reported to double in the presence of diabetes (Venugopal & Mohammed, 2014). Hypertension is known to cause damage in the microvasculature of the inner ear, similar to the damage seen in diabetes (Chao, 2004; Kakarlapudi et al., 2003). Therefore, both these conditions heighten the risk of acquiring hearing loss. This variation in the reporting of hearing loss prevalence is a potential reason why hearing impairment has not been considered a definite complication of diabetes despite the abundance of scientific evidence available.

Hearing impairment is a significant global public health issue, affecting approximately 430 million people (WHO, 2021). Low- and middle-income countries, such as South Africa, face the most substantial impact of hearing loss (WHO, 2021). Regardless of its characteristics, hearing impairment has been associated with adverse effects such as social isolation and depression, which ultimately impact quality of life (Nordvik et al., 2019). In type 2 diabetes, the commonly identified hearing loss is comparable to presbycusis and involves a more pronounced loss at higher frequencies (Akinpelu et al., 2014; Hlayisi et al., 2018). In some cases, the lower and mid-frequencies are also affected, and hearing loss has been reported to be bilateral and sensorineural (Bhaskar et al., 2014; Mozaffari et al., 2010; Ren et al., 2017). Although authors such as Agarwal et al. (2009), Krishnappa and Naseeruddin (2014) and Bhaskar et al. (2014) believe that the causes of hearing loss present in type 2 diabetes could be multifactorial, there appears to be more evidence pointing to microangiopathy of the inner ear as a potential contributor to auditory abnormalities in this patient population (Helzner & Contrera, 2016). Therefore, the permanent nature of hearing loss and its consequent effect on patient quality of life highlight the necessity of including audiological services in diabetes care.

Recent research reports on diabetes-related hearing impairment emphasise the importance of various audiological modalities for assessing and monitoring hearing in individuals with type 2 diabetes mellitus (DM). High frequency audiometry has been favoured as a method for early identification of hearing loss in patients with diabetes (Akinpelu et al., 2014; Kakarlapudi et al., 2003; Ozkurt et al., 2016) because of diabetes being associated with high-frequency hearing loss. This form of audiometry is valuable in detecting hearing loss at a subclinical phase (Valiente et al., 2014). Objective testing methods such as the Distortion Product Otoacoustic Emissions test (DPOAE) and the auditory brainstem response test (ABR) (Meena et al., 2016) have also shown value in detection of hearing impairment for patients with diabetes. The complexity of this metabolic disease, resulting in various forms of neuropathies in the body, including neural hearing loss (Feldman et al., 2019) further supports the need for a comprehensive test battery approach when assessing hearing loss in persons with diabetes. Also, the lack of universally acceptable protocol suggested for this population provides an impetus to profile hearing loss for persons living with type 2 diabetes.

Based on the available literature, it is evident that there is a high prevalence of type 2 DM in South Africa, which is known to be associated with hearing loss resulting from cochlear damage. Current studies in South Africa have been limited to peripheral auditory assessment (Hlayisi et al., 2018; Pillay et al., 2021). However, literature suggests a neurological impact associated with diabetes (Farmaki et al., 2020). Therefore, the study aimed to investigate the audiological profile of patients with type 2 diabetes in South Africa using a comprehensive audiological assessment that reflects the integrity of the entire auditory pathway.

### Study aim

To describe the audiological profile of adults with type 2 diabetes between the ages of 18 and 55 years at a district hospital in Mpumalanga.

### Study objectives

- To describe self-reported audiological symptoms in participants with type 2 DM.
- To describe the audiological profile of participants with type 2 DM in terms of type, degree, configuration, symmetry and laterality.
- To describe the auditory brainstem functioning of participants with type 2 DM.

## Research methods and design

### Research design

A quantitative, non-experimental, descriptive research design was employed to achieve the study’s objectives and address the research problem. This design involves describing and interpreting a particular phenomenon without any intrusion from the researcher (Aggarwal & Ranganathan, 2019).

### Study setting

This study was conducted at a district hospital in Mpumalanga province. This site was selected based on its functional multidisciplinary diabetes and hypertension clinic at the time of data collection, allowing easy access to participants.

### Study sample

Patients attending the hospital outpatient department were recruited using purposive sampling (Campbell et al., 2020), such that participants between the ages of 18 and 55 years who had been diagnosed with type 2 diabetes for less than 10 years were included in this study. In contrast, participants presenting with the following were excluded:

- having a family history of hearing loss,
- history of middle ear infections,
- presence of immune compromising diseases such as HIV/AIDS,
- history of ototoxic medication usage,
- exposure to recreational or occupational noise for a period of 24 h.

### Data collection

Upon receiving ethical approval as well as obtaining informed consent from the participants, medical records were evaluated to solicit information such as the type of diabetes, control status (HbA1c) of the diseases and medication consumption (Supplementary file 1). Additionally, a structured questionnaire was utilised to gather data on self-reported audiologic symptoms, hearing history, medical history and history of noise exposure (Paken et al., 2017). Subsequently, all participants underwent a series of audiological assessments, which included otoscopic examination, tympanometry, pure tone air and bone conduction, extended high-frequency (EHF) audiometry, speech reception threshold (SRT) testing, DPOAE testing and neurological ABR testing (Supplementary file 2).

### Data analysis

Prior to statistical analysis, the audiological data were evaluated against normative data (Supplementary file 2). The data in this study were described using frequencies, ranges, percentages, means, medians and standard deviations. Participants’ thresholds were analysed separately for their right and left ears to report on the degree, type and configuration of hearing loss; thereafter, the laterality and symmetry were analysed per participant. The EHFs were analysed by comparing the current study’s participant EHF medians at various age groups with those of age-appropriate normative values from Valiente et al. (2014) using the Wilcoxon signed-rank rest. The Wilcoxon rank-sum test was utilised to compare continuous patient demographics and clinical factors, while Fisher’s exact test was used for categorical variables associated with hearing loss. A two-tailed value of *p* < 0.05 was considered statistically significant. The results are presented in tables, graphs and figures. All statistical analyses were conducted using Statistical Package for the Social Sciences (SPSS) version 28. IBM^®^ SPSS^®^ Statistics, IMB Corporation, Armonk, New York, United States.

### Reliability and validity

The reliability of the results was maintained by employing standard audiological tests and procedures frequently utilised in clinical practice (Davies, 2016). These tests were conducted using equipment calibrated per South African National Standards (SANS) standards, and the researcher performed biological calibrations daily. The cross-check principle was utilised during audiological test evaluations, where each assessment measure was cross-checked with the others (Jerger & Hayes, 1976). This approach was intended to reduce the occurrence of false-positive findings, thereby improving the accuracy of the results (Turner, 2003).

### Ethical considerations

This study was guided by the ethical principles outlined by the World Medical Association (WMA), in the Declaration of Helsinki (World Medical Association, 2013). Ethical approval was granted by the Institution Ethics Committee (Protocol Number: BREC/00000835/2019). Permission to conduct data collection at the study site was obtained from the Mpumalanga Department of Health (Protocol Number: MP_202001_008). Written informed consent was sought through the participants prior to participation in the study.

## Results

### Participants’ characteristics

The study sample comprised 35 adults, 15 males and 20 females (Table 1). The participants ranged from 21 to 55 years, with a mean age of 41.03 years (standard deviation [s.d.] = 8.03). Most of the participants (85.7%) had poorly controlled diabetes, and a significant proportion (97.1%) were initiated on medication, while a smaller proportion of the participants (20.0%) presented with associated comorbidities such as hypertension.

**TABLE 1:** Participant demographic and clinical features of type 2 diabetes mellitus.

Characteristic	*n*	%
**Age (years)**		
21–30	2	5.7
31–40	18	51.4
41–50	6	17.1
51–55	9	25.7
**Gender**		
Male	15	43.0
Female	20	57.0
**Ethnicity**		
Black African people	34	97.1
White African people	1	2.8
**Control status (HbA1c)**		
Good control: < 7.0% (< 53 mmol/mol)	5	14.3
Poor control: > 7.0% (> 58 mmol/mol)	30	85.7
**Treatment regimen**		
Oral hypoglycaemic agent	34	97.1
Insulin	1	2.9
**Comorbidities**		
Hypertension	7	20.0
None	28	80.0
**Duration of diabetes**		
< 12 months (newly diagnosed)	11	31.4
1 to 4 years	18	51.4
5 to 10 years	6	17.1

HbA1c, glycated haemoglobin.

### Self-reported audiological symptoms

The study found that 9 of the 35 participants (25.7%) reported self-perceived hearing difficulties. Among those who reported hearing concerns, three participants (33.3%) reported difficulties in the left ear, while six (66.7%) reported bilateral involvement. Eight participants (88.9%) reported experiencing hearing challenges for less than 5 years. The onset of hearing concerns was reported to be gradual by six participants (66.7%). Eight participants (22.9%) reported experiencing difficulties hearing in the presence of background noise. Four participants (11.4%) reported experiencing high-frequency tinnitus. Other otologic symptoms reported were otalgia (5.7%) and dizziness (17.1%). Twenty-three (65.7%) participants presented with no associative otologic symptoms. Self-reported audiological and otologic symptoms of participants are shown in Table 2.

**TABLE 2:** Self-reported audiological and otologic symptoms.

Characteristic	*n*	%
Hearing difficulties	9	25.7
No hearing difficulties	26	74.3
Associated ear (left ear)	3	33.3
Associated ear (right ear)	0	0.0
Associated ear (both)	6	66.7
**Duration of hearing difficulties (years):**		
> 10	1	11.1
< 5	8	88.9
**Onset of hearing difficulties:**		
Sudden	2	22.9
Gradual	6	66.7
Fluctuating	1	11.1
**Associated listening situation:**		
Background noise	8	22.9
No difficulties	27	77.1
**Otologic symptoms:**		
Otalgia (earache)	2	5.7
Dizziness	6	17.1
Tinnitus: High frequency	4	11.4
No otologic symptoms	23	65.7

### Hearing loss prevalence, type, degree, configuration and laterality

The study found a 31.4% (22 ears) prevalence of hearing loss among participants. Fisher’s exact test showed no statistically significant gender difference in hearing loss prevalence (*p* = 0.07). Among the 22 ears with hearing loss, 18 (81.1%) had sensorineural hearing loss. Slight hearing loss was the most common degree of hearing loss, accounting for 36.4% of cases. A rising configuration was observed in 36.4% of participants. Bilateral hearing loss involvement was observed in 10 participants (83.3%), and 6 participants (60%) presented with an asymmetrical hearing loss (Table 3).

**TABLE 3:** Type, degree, configuration, laterality and symmetry.

Description	*n*	%
Total sample size	35	100.0
Total number of ears in sample	70	100.0
Number of ears with normal hearing	48	68.6
Number of ears with hearing loss	22	31.4
**Type of hearing (ears, *n* = 22)**		
Conductive	1	4.5
Sensorineural	18	81.8
Mixed	3	13.6
**Degree of hearing loss† (ears, *n* = 22)**		
Slight	8	36.4
Mild	3	13.6
Mild to moderate	4	18.2
Moderate	1	4.5
Moderate to moderate-severe	1	4.5
Moderate to severe	2	9.0
Moderate severe to severe	1	4.5
Moderate to profound	1	4.5
Profound	1	4.5
**Configuration (ears, *n* = 22)**		
Sloping	7	31.8
Rising	8	36.4
High frequency	5	22.7
Flat	2	9.0
**Laterality (participants, *n* = 12)**		
Bilateral	10	83.3
Unilateral	2	16.6
**Symmetry (participants, *n* = 12)**		
Symmetrical	4	40.0
Asymmetrical	6	60.0

† , As cited in Clark (1981).

### Glycaemic control status and hearing status

Fischer’s exact test revealed no statistically significant relationship between HbA1c and hearing status for the right ear (*p* = 0.9) and the left ear (*p* = 0.64), thus indicating that the control status of diabetes did not influence hearing.

### Type 2 diabetes, comorbidities (hypertension) and hearing status

A statistically significant relationship existed between hypertension and hearing loss in the right (*p* = 0.006) and left (*p* = 0.02) ears. These findings suggest that hypertension increases the risk of hearing loss. However, we did not observe any statistically significant relationship between the duration of diabetes and hearing loss in either the right (*p* = 0.44) or left (*p* = 0.386) ear.

### Self-reported audiologic and otologic symptoms with hearing loss

The study found that individuals who reported difficulty in hearing were found to have hearing loss with statistically significant results for both right (*p* = 0.003) and left ears (*p* ≤ 0.001). However, there was no statistically significant difference in the proportion of different symptoms (i.e. earache, tinnitus, dizziness) reported between the two ears, that is, right ear (*p* = 0.19) and left ear (*p* = 0.36). Table 4 depicts the relationship between self-reported otologic and audiologic symptoms and hearing status of participants.

**TABLE 4:** Relationship between self-reported otologic and audiologic symptoms and hearing status of participants.

Characteristic	Normal hearing		Hearing loss		*p*
	*n*	%	*n*	%	
**Right ear**					
Earache	2	7.7	0	0.0	0.190
Tinnitus	2	7.7	3	33.3	-
Dizziness	18	69.2	4	44.4	-
Self-perceived hearing loss	3	11.5	6	66.7	0.003*
**Left ear**					
Earache	1	4.2	1	9.1	0.360
Tinnitus	2	8.3	3	27.3	-
Dizziness	17	70.8	5	45.5	-
Self-perceived hearing loss	1	4.2	8	72.7	< 0.001*

* , The data was highly significant (*p* < 0.05).

### Otoscopic examination

Among 22 ears with clinically confirmed hearing loss (Newsted et al., 2020), the otoscopic examination was normal for the right and left ear (*n* = 22; 100%).

### Tympanometry

Of 22 ears with hearing loss, 18 (81.1%) presented with type A tympanograms; followed by 2 ears (9.1%) with type As and 2 ears (9.1%) with type Ad tympanograms.

### Pure tone audiometry

**FIGURE 1:**
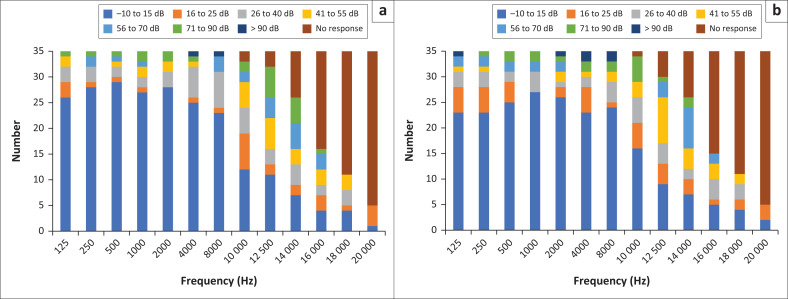
(a) Distribution of participants’ air conduction thresholds within the various intensity ranges at the different frequencies for the right ear. (b) Distribution of participants’ pure tone air conduction thresholds within the various intensity ranges at the different frequencies for the left ear.

Conventional pure tone audiometry (250 Hz – 8000 Hz) and EHF audiometry (10 kHz; 12.5 kHz; 14 kHz; 16 kHz; 18 kHz and 20 kHz) were conducted. In the right ear, a large proportion of participants presented with no responses at frequencies of 16 kHz (*n* = 19; 54.3%), 18 kHz (*n* = 24, 68.6%) and 20 kHz (*n* = 30, 85.7%). Similar findings were also noted in the left ear at 16 kHz (*n* = 20; 57.1%), 18 kHz (*n* = 24; 68.6%) and 20 kHz (*n* = 30; 85.7%). It appears that as the frequency increases, so too does the hearing loss. Therefore, this implies high frequencies are most affected. Figure 1 depicts the distribution of participants’ pure tone thresholds within the various intensity ranges at the different frequencies for the right and left ear.

### Extended high frequencies

**FIGURE 2:**
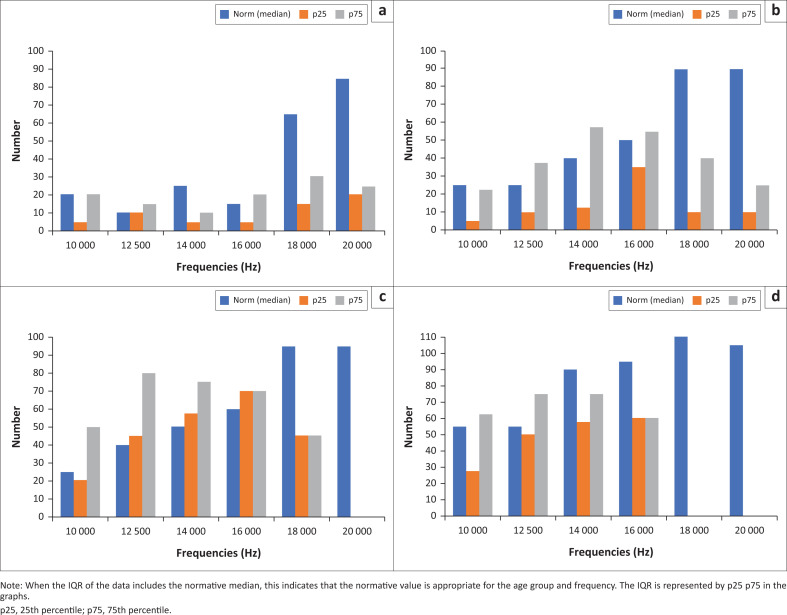
Comparison between the extended high frequency interquartile range (IQR) for various age groups with the age-appropriate normative data (right ear). The age groups include: (a) 20–29 years; (b) 30–39 years; (c) 40–49 years and (d) 50–59 years.

**FIGURE 3:**
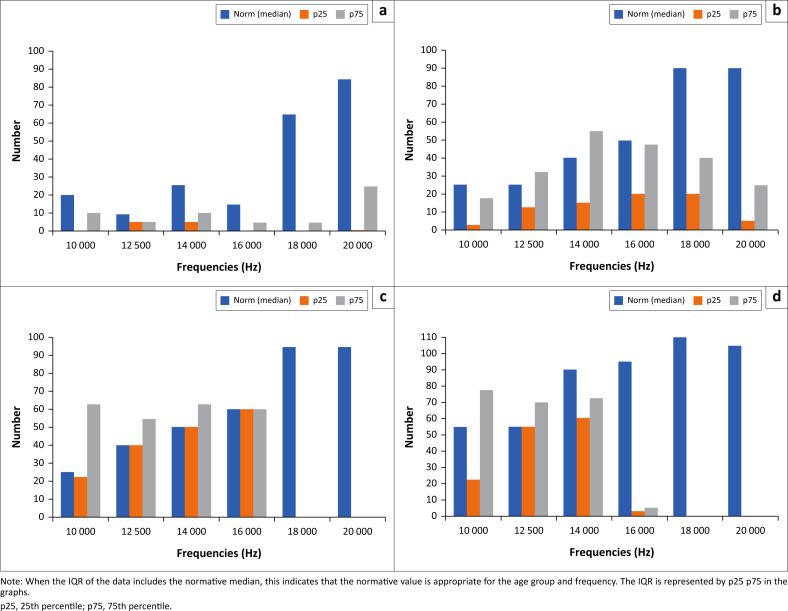
Comparison between the extended high frequency interquartile range (IQR) for various age groups with the age-appropriate normative data (left ear). The age groups include: (a) 20–29 years; (b) 30–39 years; (c) 40–49 years and (d) 50–59 years.

This study evaluated the extended high frequencies (EHF) to identify subclinical hearing impairment. The results presented in Figure 2 and Figure 3 illustrate a comparison of EHF medians for different age groups in the right and left using age-appropriate normative values from Valiente et al. (2014).

**FIGURE 4:**
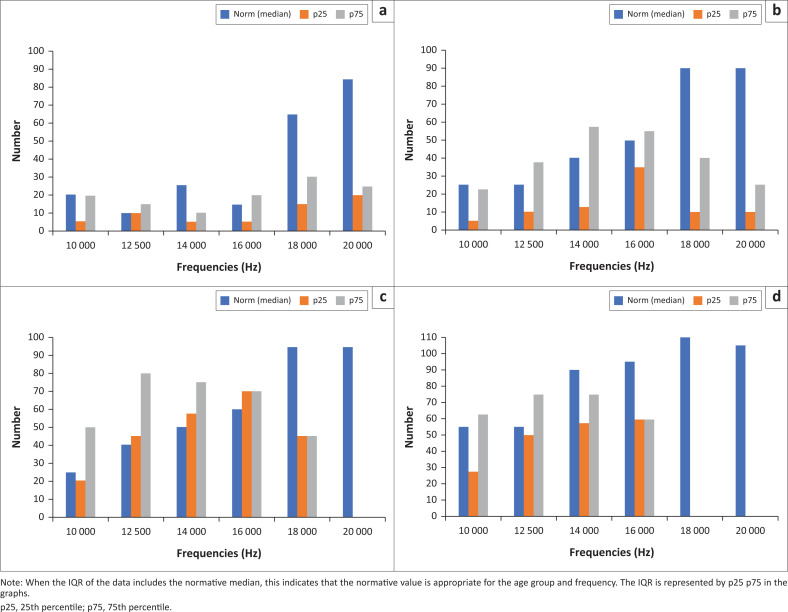
The comparison between participants’ medians and age-appropriate norms. (a) 20–29 years; (b) 30–39 years; (c) 40–49 years and (d) 50–59 years.

The Wilcoxon signed-rank test was utilised to compare the median EHF with age-appropriate normative values (Valiente et al., 2014). The comparison between all medians was not statistically significant from the normative values for both the left and right ear, as depicted in Figure 4. This is because, from 16 kHz and above, the age appropriate norms exceed the interquartile range (IQR) of the medians for all ages. This could be attributed to the sample size and fewer observations at levels above 16 kHz in the age group above 50 years.

When the IQR of the data includes the normative median, this indicates that the normative value is appropriate for the age group and frequency. The IQR is represented by p25 p75 in the graphs. In Figure 4, this occurs in only 50% of the age groups and is primarily in the lower EHF frequencies, and this is consistent across all age groups.

#### Speech reception threshold

The SRT was used to ascertain the reliability of pure tone results. Of 22 ears with hearing loss, 11 (50%) presented with good SRT-PTA correlation in the right ear and 9 (40.90%) in the left ear. A fair correlation was observed in one ear (4.54%), the right ear, and similar observations were noted for the left ear.

#### Distortion Product Otoacoustic Emissions Test

Normal DPOAEs were present bilaterally across the 1000 Hz – 4000 Hz frequency range. However, absent DPOAEs were observed at 6 kHz (*n* = 20; 51.7%) and 8 kHz (*n* = 24; 68.6%) in the right ear. A similar pattern was observed in the left ear, with absent DPOAEs being recorded in most participants at 6 kHz (*n* = 17; 48.6%) and 8 kHz (*n* = 29; 82.9%). These findings suggest that high-frequency DPOAEs are affected first.

#### Auditory brainstem response results

Delayed absolute latency of wave III was seen in both the right ear (*n* = 24, 69%) and the left ear (*n* = 18, 51%). Fewer participants had delayed absolute latency of wave V in the right (*n* = 10, 29%) and left ears (*n* = 6, 17%). However, most participants had normal absolute latency of wave I in the right ear (*n* = 30, 85.7%) and left ear (*n* = 23, 65.7%). Interpeak latency delay of waves I-III was present in the right ear of 12 participants (34.3%) and the left ear of 10 participants (28.6%). Conversely, early interpeak latency was noted for waves III-V in the right ear (*n* = 13) and the left ear (*n* = 15), which in this case would be considered to be within normal limits in light of the delayed absolute latency of wave III. Similar findings were observed for waves I–V bilaterally. This finding suggests that ABR is sensitive to subtle neural dys-synchrony that standard pure-tone audiometry might not have detected.

## Discussion

The study found that 25.7% (*n* = 9) of our participants reported bilateral self-perceived hearing difficulties, which is higher than Soares et al. (2018), who indicated a prevalence of self-perceived hearing impairment of 6.69% in their diabetic population. This variation in self-perceived hearing impairment findings could be attributed to the fact that Soares et al. (2018) found that age, race, education, occupational exposure, smoking and alcohol consumption were positively associated with self-reported hearing impairment. In contrast, the current study could not account for the impact of such variables in the findings. There was a lower prevalence of tinnitus (11.4%) among participants with confirmed hearing loss compared to previous reports of 68.7% (Berner et al., 2017) and 45.1% (Pillay et al., 2021). A possible reason for the lower prevalence of tinnitus in the current study is that 51.4% (*n* = 18) of the participants had diabetes for less than 5 years. This could have possibly indicated reduced microvascular complications such as those associated with tinnitus.

In light of self-perceived audiological and otologic symptoms, there was a lower prevalence of dizziness (17.1%) compared to previous reports by Nemati et al. (2018) and Pillay et al. (2021), who reported higher percentages of 26.0% and 42.4%, respectively. However, caution should be exercised when interpreting these findings, as these studies could not distinguish dizziness resulting from inner ear defects or glucose alterations. A comprehensive vestibular assessment is necessary to make this distinction. Regrettably, this was beyond the scope of the current study.

The prevalence of hearing loss among individuals with type 2 diabetes varies across regions of the world (Bhaskar et al., 2014; Hlayisi et al., 2018; Kakarlapudi et al., 2003). The present study found the prevalence of hearing loss in adults with type 2 diabetes to be 31.4%, which is lower than that reported in the literature (Dhasmana et al., 2021; Hlayisi et al., 2018; Idugboe et al., 2018; Mozaffari et al., 2010; Pillay et al., 2021). However, the current findings are consistent with the prevalence rate of 21.6% reported in Nigeria by Adebola et al. (2016). It should be noted that the comparison of prevalence rates is complex, as it is influenced by various factors, such as the type of assessment protocols employed (diagnostics vs. screening), study sample compositions and normative data utilised. For example, Pillay et al. (2021) and Bashkar et al. (2014) used the pure tone average (PTA) to determine the prevalence of hearing loss, and Hlayisi et al. (2018) utilised low-frequency PTA and high-frequency PTA cutoffs, while the current study took all frequencies into account (125 Hz-8 kHz) when determining the degree of hearing loss. Regardless of this variation in the methods of establishing the prevalence of hearing loss across the literature, the present study still confirms the existence of hearing loss in type 2 diabetes and emphasises the importance of incorporating audiological assessments in the routine care of these patients.

Hearing loss can be categorised into three types: conductive, sensorineural or mixed (Alshuaib et al., 2015). Of 22 ears with hearing loss, 81.1% had sensorineural hearing loss. This finding is consistent with previous literature reports indicating that sensorineural hearing loss is the most common type of hearing loss observed in persons with type 2 diabetes (Hlayisi et al., 2018; Idugboe et al., 2018; Pillay et al., 2021; Samocha-Bonet et al., 2021). The pathophysiological basis of sensorineural hearing loss specific to DM may be because of microangiopathy of the inner ear, neuropathy of the cochlear nerve or a combination of both factors, as suggested in the literature (Kakarlapudi et al., 2003). Further support for this theory comes from the study conducted by Fukushima et al. (2005), who noted anatomic changes in the cochlear structures of insulin and non-insulin diabetic patients. Evidence from Makishima and Tanaka (1971), though dated centuries ago, still provides insightful knowledge regarding the auditory anatomic defects occurring in persons with diabetes. The authors identified atrophy of spiral ganglia in the basal to middle turns of the cochlea and demyelination of the VIII Cranial nerve myelin sheaths (Makishima & Tanaka, 1971), thus ultimately affecting the physiological aspect, which is hearing, potentially resulting in neural hearing loss. These findings highlight the multifactorial nature of hearing impairment in diabetes and the need for early detection and management of hearing loss in people with diabetes.

The current study observed slight hearing loss (16 dB – 25 dB) in 36.4% of the participants, consistent with the findings of Hlayisi et al. (2018), who reported a similar rate of 35.0% in their diabetic cohort (Hlayisi et al., 2018). Minimal or slight hearing loss is used to describe hearing thresholds within 16 dB – 25 dB (Kaderavek & Pakulski, 2002; Martin & Clark, 2003). Despite its subtle nature, slight hearing loss can negatively impact speech reception, discrimination and comprehension, as previously documented in the literature (Arlinger, 2003). Moreover, hearing loss has been associated with various adverse outcomes, including social isolation, depression and cognitive decline (Croll et al., 2021; Kim et al., 2023). Recently, there has been growing evidence linking hearing loss with dementia (Griffiths et al., 2020), highlighting the importance of early detection and management of hearing loss to minimise the negative consequences accompanying hearing loss.

In this study, the most common hearing loss configuration observed among individuals with diabetes was rising (36.0%) and sloping (31.8%). To our knowledge, there is a lack of studies that have specifically investigated and reported the configuration of hearing loss in diabetes (Al-Rubeaan et al., 2021). Understanding the specific configuration of hearing loss in diabetes may improve intervention outcomes, such as those requiring amplification.

In the literature, high-frequency involvement has been reported as a hallmark feature of diabetes-related hearing impairment (Meena et al., 2016; Ozkurt et al., 2016). The current study also found high-frequency hearing loss in 22.7% of cases. However, this prevalence rate is lower than those reported by Hlayisi et al. (2018) and Ren et al. (2017), who found high-frequency hearing loss in 55% to 72.2% of cases. While the previous studies of Hlayisi et al. (2018) and Ren et al. (2017) determined high-frequency loss by averaging frequencies of 4 kHz, 6 kHz and 8 kHz, the current study did not utilise frequency averages when determining the prevalence. The analysis took the frequency range of 125 Hz to 8 kHz into account when determining the degree of hearing loss, which aligns with the hearing loss classification scheme from the ASHA (1981), which may explain why the prevalence is perceived as low.

To further peruse hearing loss beyond the frequency range routinely tested in clinical practice, we employed EHF testing (> 8 kHz to 20 kHz), which is more sensitive to subclinical hearing loss than conventional audiometry (Valiente et al., 2014). The results showed that as the frequency increased, the hearing loss also increased, with most participants showing ‘no responses’ at frequencies of 16 kHz, 18 kHz and 20 kHz. These findings are consistent with those of Ozkurt et al. (2016) and Nemata et al. (2018), who observed increased hearing loss as the frequency increased. As seen in diabetes, the higher hearing frequencies are reported to be affected first before the standard hearing frequency range is affected (Das et al., 2018). Hearing loss in the higher frequencies (> 4000 Hz) can have a negative impact on the perception of certain speech sounds, like ‘f’, ‘s’ and ‘th’, including sounds perceived in nature and music (Paken et al., 2023). However, the detection of sounds between 5000 Hz and 9000 Hz is important for humans as it ensures proper acoustic perception as they ‘guarantee a good part of speech intelligibility by favouring consonant discrimination and speech recognition’ (Anastasio et al., 2012, p. 39). Therefore, this finding further favours EHF audiometry – a valuable measure in this patient population. Additionally, the results provide tangible evidence that EHF hearing thresholds could be considered a potential sensitive marker of hearing loss in type 2 diabetics.

This study utilised the SRT test to determine the validity of pure tone audiometry. The majority of the ears with hearing loss showed a good correlation between SRT and pure-tone average (PTA), which indicates reliable pure-tone results (Table 5). A correlation of less than 5 dB was considered good, a correlation between 6 dB and 9 dB was considered fair and a correlation greater than 10 dB was considered poor (Gelfand, 2001). Though this study found a good SRT-PTA correlation, Kiakojouri et al. (2014) reported worse SRT in their diabetic group compared to the control group. There seems to be no current evidence reporting SRT outcomes in diabetic patients.

**TABLE 5:** Speech reception threshold test (SRT) and pure-tone correlation (PTA).

Puretone testing reliability	Right ear		Left ear	
	*n*	%	*n*	%
Good SRT-PTA	11	50.00	9	40.90
Fair SRT-PTA	1	4.54	1	4.54
Poor SRT-PTA	0	0.00	0	0.00

**Total**	**12**	**54.54**	**10**	**45.44**

Previous studies have investigated various patient characteristics associated with diabetes-related hearing impairment, including diabetes control status, duration and associated comorbidities (Al-Rubeaan et al., 2021; Hlayisi et al., 2018; Pillay et al., 2021). The present study examined the impact of hypertension on hearing loss in persons with type 2 diabetes. In hypertension, adverse synergistic effects with inner ear microvascular damage resembling damage caused by diabetes have been observed (Chang et al., 2011; Chao, 2004; Kakarlapudi et al., 2003). From a physiological point of view, the combined presence of hypertension and diabetes is believed to lead to damage in the cochlea, thus resulting in high-frequency sensorineural hearing loss (Duck et al., 1997). Recognising the risk profile can enhance the understanding of how these risk factors interplay in type 2 diabetes. Simultaneous exposure to multiple risk factors such as co-morbidities (i.e. hypertension) can exacerbate auditory dysfunction more than if each factor was considered separately, thus implying synergistic effect (Chao, 2004; Paken et al., 2021). This may be plausible in this study, as a statistically significant association between hypertension and hearing loss was observed in both the right ear (*p* = 0.006) and the left ear (*p* = 0.02).

In Nigeria, Babarinde et al. (2021) found a statistically significant association between hypertension and hearing loss (*p* < 0.010) in their study of 500 individuals, which included a control group. Hearing loss in hypertension was further reported to be mild in degree, sensorineural in nature, as well as increased with age, severity and duration of the disease. This study’s findings and previous research suggest that comorbidities such as hypertension, when present with type 2 DM, may increase the risk of acquiring hearing impairment, thus implying a synergistic effect.

Previous research has consistently reported that the duration of diabetes impacts the development of sensorineural hearing loss (Samocha-Bonet et al., 2021). However, the current study did not find a statistically significant association between diabetes duration and hearing impairment. The researchers postulate that the lack of statistical significance may be attributed to the observed relatively lower prevalence rate of hearing loss because of the small sample size. Further studies with a larger sample size and a wider range of diabetes durations are needed to confirm this hypothesis. The researchers speculate that this lack of association in the present study could also be attributed to a large proportion (88.9%) of the participants having diabetes for less than 5 years. It is also possible that a longer duration of diabetes may be necessary for the development of hearing impairment, which emphasises the need for glucose monitoring.

The auditory system depends on glucose for energy and functioning (Elibol & Baran, 2020). Monitoring glucose levels is crucial for persons with diabetes to prevent diabetes-related complications. Previous studies have established a link between poor glucose control and hearing loss in persons with diabetes (Al-Rubeaan et al., 2021; Elibol & Baran, 2020). However, the current study did not find a significant statistical association between glycaemic control status and hearing impairment, even though most participants (85.7%) had poor glycaemic values, indicating poor control of the disease. In contrast, Al-Rubeaan et al. (2021) reported that poor glycaemic control of > 8% was associated with hearing loss in their study. Although this study could not establish a statistically significant relationship, there is still abundant research showing the influence of poor glycaemic control on the auditory acuity of diabetes patients (Ebol & Baran, 2020; Nagahama et al., 2018; Srinivas et al., 2016). Audiologists are urged to emphasise the importance of glucose monitoring in their counselling or education with diabetes patients to prevent the possible occurrence of hearing impairment or worsening of hearing impairment when present.

There is scientific evidence suggesting that patients with diabetes may experience damage in the outer hair cells (OHCs) of the cochlea (Fukushima et al., 2005; Hlayisi et al., 2018). It is postulated that the dysfunction of OHCs in diabetic patients could be attributed to disruptions in glucose metabolism (Kumar et al., 2021). Glucose is recognised as a potential energy source for the cochlea (Kumar et al., 2021). Therefore, any disturbances in glucose metabolism could potentially impact the proper functioning of OHCs. The present study revealed that most participants had absent DPOAE in the frequency range of 6 kHz – 8 kHz bilaterally, suggesting that diabetes impacts the higher frequencies more. The loss of OHCs within the frequencies mentioned above could imply that the impact is more on the region of basal turns of the cochlea (Fukushima et al., 2005). Consistent with the current findings of OHCs dysfunction are Ferreira et al. (2016) and Hlayisi et al. (2018), who reported a high prevalence of absent OAEs in diabetic patients (Hlayisi et al., 2018; Selvarajah et al., 2011). These findings emphasise the importance of incorporating cochlear monitoring using measures such as DPOAE testing and that DPOAEs are sensitive in detecting high-frequency hearing loss in this patient population.

One common complication in individuals affected by diabetes is peripheral neuropathy (Feldman et al., 2019). Other studies have reported the presence of central neuropathy in diabetic patients (Selvarajah et al., 2011). The current study utilised ABR testing to assess the integrity of the auditory neural system. The findings reveal that most participants exhibited delayed absolute latency of wave III in both the right ear (*n* = 24, 69%) and the left ear (*n* = 18, 51%), which is consistent with the findings reported by Siddiqi et al. (2013) who utilised a neurological protocol in their testing. The high proportion of delayed absolute latency of wave III observed in this study suggests a possible lesion in the superior olivary complex (SOC). The SOC is an essential site of auditory stimuli convergence from both ears and plays a significant role in sound localisation (Grothe et al., 2010). Therefore, the observed abnormalities in wave III latency may affect the perception and processing of sound localisation cues in individuals with diabetes-related hearing impairment. This finding supports the assertion that ABR is sensitive to subtle auditory neural changes in diabetes. To the researcher’s knowledge, currently in the South African published literature regarding diabetes-related hearing impairment, the current study has pioneered the comprehensive audiological evaluation achieved by incorporating ABR in the assessment of diabetic patients. It has provided insight into the auditory neural integrity of diabetic patients. Although the findings advocate for the inclusion of ABR in audiologic test batteries for diabetes, this measure may not be economical for low- and middle-income countries because of the costs associated with procuring and maintaining such equipment.

### Implications

This study has revealed important implications for clinical practice, healthcare practitioners, academic community, as well to policy makers. The implications derived from this study were:

- Clinical practice and patient care:
  The current finding challenges a paradigm shift in the audiologic assessment and management practices by recognising the impact of non-communicable diseases (NCDs) like type 2 diabetes on the hearing system. Knowledge from the current study also challenges healthcare providers involved in diabetes care to refer all diabetic patients for hearing evaluation to enable early detection and timely intervention of auditory-related complications.
- Audiological assessment protocol:
  In terms of audiological clinical testing protocols, the study suggests the following:
- A tailored case history questionnaire should be utilised to solicit information about self-reported symptoms such as subjective hearing loss, tinnitus, dizziness and vertigo resulting from inner ear defects.
- Pure tone audiometry, using strict hearing loss classification criteria (< 16 dB), is recommended to detect subtle hearing loss, such as slight hearing loss, which was evident in the current study, as well as in previous findings by Hlayisi et al. (2018).
- Extended high-frequency audiometry can help identify high-frequency hearing loss at a subclinical stage.
- Diagnostic DPOAEs with a targeted diagnostic audiometry protocol is suggested for early detection of cochlear damage before it becomes overt in conventional audiometry.
- Lastly, using ABR testing, specifically a neurological protocol, though not economical to resource-constrained countries like South Africa, would allow audiologists to identify subtle neural dys-synchrony before reaching clinical manifestation.
- Awareness and education:
  The study findings can inform educational initiatives for both healthcare professionals and patients. Knowledge from this study can be incorporated in already existing diabetes educational programmes such as the known International Diabetes Federation (IDF) school of diabetes, which reaches both healthcare providers and patients in masses. Knowledge from this study can also inform higher education health sciences curriculum to consider including potential auditory consequences associated with diabetes when engaging with teachings of NCDs like diabetes. Such educational initiatives will increase awareness about the audiological risk associated with type 2 diabetes and subsequently, will foster early detection and prevention of audiological risks present in diabetes.
- Multidisciplinary collaboration:
  This study advocates for increased collaboration among diverse healthcare professionals, encompassing audiologists, endocrinologists, primary care physicians, nurses and health promoters, to strengthen the interdisciplinary care provided to individuals with diabetes and concurrently address audiological concerns and possibly increase referrals to the audiologist. Diabetes mellitus, a complex metabolic disorder, has been associated with an elevated risk of auditory complications. By promoting collaborative efforts, there would be improved patient outcomes through a comprehensive and integrated healthcare approach.
- Policy and resource allocation:
  Knowledge from this study challenges policy makers to allocate both human and equipment audiological resources into the already existing healthcare system to address the audiological concerns resulting from this NCD. The findings may also argue for the inclusion of hearing impairment as a comorbidity of type 2 diabetes into existing policies such as the National Department of Health guideline on the management of type 2 DM at the primary care level (National Department of Health, 2014) as well, the guideline of the Society for Endocrinology, Metabolism and Diabetes of South Africa (SEMDSA), of which both guidelines speak to the diagnostics, management and preventative aspects of diabetes.
- Public health campaigns:
  The findings can be utilised for public awareness campaigns to increase awareness and access to knowledge, which may ultimately influence self-help-seeking behaviours. This can be achieved by incorporating diabetes-related hearing impairment knowledge into public awareness tools, like the diabetes fact sheet published by the IDF and the World Health Organization (WHO). Awareness campaigns can be aimed to promote regular audiological check-ups for individuals with diabetes. The findings may also be utilised to encourage audiologists serving at a primary health care level to be involved in non-communicable programmes such as that of diabetes, providing health talks regarding diabetes and hearing loss as a method of increasing awareness.
- Quality of life and well-being:
  Persons living with diabetes are faced with an array of complications, which may alter the state of their quality of life and wellbeing. Hearing impairment on its own has been associated with poor quality of life resulting from isolation, limited social participation and communication deficits. Knowledge from this study encourages healthcare providers to recognise the magnitude of the impact of these conditions on the patient’s wellbeing and refer for appropriate intervention such as psychology.
- Continuing professional development:
  This study’s findings encourage ongoing professional development for audiologists, particularly in the context of diabetes care. Continuing professional development would enable audiologists to stay up to date latest trends and best practices. Not only would it expand knowledge for the health professional, but it will broaden the professional’s skill set and improve clinical care.
- Long-term monitoring:
  One of the classic features of hearing impairment in diabetes is that it is progressive in nature. Knowledge from this study suggest inclusion of audiological tests such as EHF audiometry and DPOAE within a target protocol as an early identification and monitoring tools for hearing impairment in the context of diabetes. There is, however, a need for a study that will investigate the hearing loss monitoring interval of these patients.
- Health equity and access:
  Knowledge from this study increase access to audiological services to persons living with diabetes. This would imply that every person living with diabetes will have a fair opportunity to attain the highest level of healthcare. This study promotes health equity and access by generating evidence that encourages the inclusion of audiological services in routine diabetes care.

### Recommendations

The study’s findings can assist to expose areas or gaps for further research in audiology and diabetes management. It encourages researchers to explore specific aspects of audiological profile and diabetes, potentially leading to interventions and therapies. Based on the study’s current findings, future research can explore studies with larger sample size and employ different methodologies other than the current one utilised in this study. Future study can thorough investigate different auditory and diabetes variables to enhance understanding about the link between this metabolic condition and hearing loss.

## Conclusion

The intricate relationship between type 2 DM and its associated complications, including hearing loss, emphasises the urgency for a holistic, multidisciplinary approach in managing this complex condition. Collaboration among healthcare professionals such as audiologists, primary healthcare nurses, endocrinologists, physicians and health promoters is essential to provide comprehensive care addressing both metabolic and audiological concerns. Audiologists play a crucial role in mitigating the adverse effects of hearing impairment in diabetes. Their involvement spans prevention, screening, diagnosis and management of auditory disorders. Employing an audiologic test battery approach guided by the cross-check principle enables early identification of auditory alterations, crucial for timely intervention. Additionally, proactive engagement in diabetes health promotion campaigns increases awareness among individuals with diabetes, influencing self-help seeking behaviour.

Diabetes and hearing loss are significant public health concerns, which necessitates attention from policymakers and health professionals. Knowledge generated from this study should inform policymakers in crafting service delivery models tailored for South Africa’s diabetic population. Strong advocacy for the inclusion of audiological services and adequate resources becomes imperative, promoting health equity and accessibility. By acknowledging the complex connection between diabetes and hearing impairment across policy and clinical realms, individuals with diabetes in South Africa stand to benefit immensely. Recognition and integration of audiological care within diabetes management promise an enhanced quality of life, ensuring timely identification, diagnosis and intervention of auditory disorders.
